# 

**Published:** 1889-04-13

**Authors:** 


### The Students' Column.

### "A TEXTBOOK OF PATHOLOGICAL ANATOMY AND PATHOGENESIS."*

*(Concluded from page 7.)*

We now cursorily refer to several subjects of interest. Among the malformations of the kidney, the condition known as "floating kidney" is mentioned as due to a peritoneal fold loosely attaching the kidney to the spine. It is more common in women than in men, in the right side than the left. Sometimes, however, a floating kidney is so movable that it appears in almost every position in the side of the abdomen to which it belongs, except the right position. This extreme mobility occasionally existing, is not mentioned by the authors. On kidney disease, it is observed that the works of Bright and Frerichs have given rise to a vast number of memoirs, of which some sixty are mentioned. There is also a summary showing how widely authorities differ as to the meaning of the term "Bright's disease." There was a time when the designation had a special significance; but at present, maladies of the kidneys have been so much more fully investigated, and so divided and subdivided, that Bright's disease does neither describe nor comprise the whole. When Bright described kidney disease, such terms as acute diffuse nephritis, acute disseminated interstitial nephritis, chronic parenchymatous nephritis, and various others, had not been coined. Bright's disease, in former days, signified almost any renal malady characterised by albumen in the urine. It is now usually held to include three different conditions—viz., inflammatory, waxy or amyloid, cirrhotic or gouty. The morbid changes are—first, inflammatory or a congested condition; next, fatty degeneration; then atrophy; and the chain of symptoms are diminution of the quantity of urine, albuminuria, often hæmaturia, tube casts, and dropsy. But the author of "Special Pathological Anatomy," instead of taking this view, has "thought it best, in dealing with the pathological anatomy of the nephritic process, to refer as little as possible to existing literature," which seems almost equivalent to ignoring Bright. Now, we object to the British pioneer of renal maladies, Dr. Bright, being in any manner ignored, for, however much our knowledge of the conditions accompanying albuminous urine has advanced since Bright was at his zenith, we know very little more about the cure of renal maladies. In the chapter on "Pyelitis" it is remarked, "irritant substances are excreted by way of the kidneys; they frequently set up inflammation in the mucus membrane of the pelvis." As we are told by morbid anatomists that inflammation of the pelvis of the kidney occurs irrespective of inflammation of other parts of the organ, we must, as in duty bound, accept the position, and may even imagine inflammation taking place when a foreign body, such as a calculus, is formed and localised on the pelvis. But when we are told that inflammation from many other causes may affect the pelvis and not the kidney, we doubt, from the close proximity and intimate connection of the parts, if this is possible. The prominent symptoms of inflammation of the kidney and of the pelvis are the same; but when the latter part is affected, the peculiar angular-tailed epithelial cells with which the pelvis is lined may generally be detected in the urine. If there are no tube casts it may be surmised there is no nephritis. But, as a very general rule, when there is pyelitis there is also nephritis, and treatment, notwithstanding pathological anatomy, should be the same. In chapter 75, "morbid changes in the supra-renal capsules" are given, which consist of caseous fibroid degeneration, often accompanied by "bronzing of the skin" and anæmia. Now, we dissent from the generally received dictum that bronzing of the skin is connected—effect and cause—with disease of the supra-renal capsules. Dr. Wilks long since showed that the supra-renal capsules may be diseased without any of that bronzing which Addison connected with such conditions, and, moreover, a similar bronzing not unfrequently occurs with the tropical malady known as *diarrhœa alba*. It seems probable that so-called "Addison's disease" is simply a form of anæmia, in which slight deposition of pigment takes place in the rete-mucusom. In the chapter on "Disorders of the Circulation in the Lung," it is stated anæmia of the lung may be due to general anæmia. It is scarcely to be supposed that anæmia of the lung, through which organ so much blood circulates, could be due to any other cause, unless the passage of the nutrient blood to the lung were cut off by organic change. Again, at pp. 144, 145, the black deposit found in the lungs in certain diseases are rather referred to particles inhaled than to pigmentation. But this dark or black colouring of the lungs presents in the inhabitants of semi-desert districts, where there are no black particles to inhale; although a sufficiency of fine sand, which, by irritating the lungs, may give rise to deposit of pigment. In the article on forms of pneumonia we find the statement that "many of the changes in the lungs set down to syphilis have really no connection with it, but are due to other causes." This may be true; but, on the other hand, many of the changes in the lungs are due to syphilis, especially in the East, where, in Europeans at least, changes in the lungs from climatic causes are less frequent, although common enough in the syphilitic subject. On tuberculous affection of the lungs the author observes that, "since the discovery by Koch of the tubercle bacillus, the question of predisposition has fallen into the background." In the last Harveian oration, Dr. Latham assigned the part of the tubercle bacillus to be that of an invader, which found a genial nidus in the persons of those living under general unsanitary conditions. We cannot, therefore, yet afford to cast predisposition into the background. The article on the etiology of goitre is interesting. The author writes that the "most probable explanation seems to be that the exciting cause of goitre is of a miasmatous nature." From this we dissent. All that can be certainly advanced as regards the causation of goitre is, that where the malady prevails lime will generally be found in the water, although the rule is not of universal application; that goitre is most prevalent in valleys at the bases of, or within the spurs of, or near lofty mountains; and that those coming down into the valleys, especially immediately after a rainy season, are most likely to be attacked.

By the foregoing observations it is far from our desire to cast the least discredit on this volume of "Special Pathological Anatomy." Everything in the powers of the learned author and editor of the English edition has been done to render the work complete, and only those who have attempted to compass a work of the kind can realise how much labour has been expended on "Special Pathological Anatomy," which, so far as relates to the signs and symptoms of disease, is almost a work on medicine also. The English translator and editor, Dr. Donald MacAlister, gives sincere thanks to Dr. James Ross, who assisted in the parts relating to the nervous system.

* "A Textbook of Pathological Anatomy and Pathogenesis." By Ernest Ziegler, Professor of Pathological Anatomy in the University of Tubingen. Translated and Edited for English students by Donald MacAlister, M.A., M.D., Fellow Royal College Physicians, University Lecturer on Medicines, and Physician to Addenbrookes Hospital, Cambridge. Part II., sect. ix.-xii. Macmillan and Co., London.

### IMPORTANT NOTICE.

### A ROYAL NUMBER

Of The Hospital will be published on April 27th, 1889. It will contain an account of the Public Life and Work of their Royal Highnesses

THE PRINCE AND PRINCESS OF WALES.

*** The monthly part for May will contain Special Portraits of their Royal Highnesses. See notice below as to separate copies.

Intending Subscribers should send their names without delay to the Publisher, The Hospital, 139-140, Salisbury Court, Fleet Street, E.C.

### Scraps and Gleanings.

There are 200 lunatic asylums in Great Britain.

There are 7,000 lunatics in the Parisian asylums.

The Queen has given £100 to the China Famine Relief Fund.

Dr. Caffyn, the inventor of liquor carnis, is now in England.

Workers on rosewood are supposed to be specially liable to eczema.

The workmen's contributions to Halifax Infirmary for 1888 exceeded £1,000.

Glasgow has sent a second instalment of £500 towards the China Famine Relief Fund.

Madame Nordica has promised to sing at a charity concert in aid of Ashford College Hospital.

On May 18 a fete and athletic competition will be held at the Homes for Little Boys, Farningham.

Baron F. von Mueller read a paper on pharmacology before the Melbourne Medical Congress.

Southampton Hospital Saturday and Sunday Fund exceeds £500 so far. Last year it reached £895.

An Institution of English Trained Nurses has been opened in the Rue de Prony, Paris, by Mrs. Levich.

On Tuesdays during May, Mr. Marcus Gunn will lecture at Great Ormond-street on "Eye Disease in Children."

After a case of erysipelas at Ashford Cottage Hospital, the sum of £42 was spent in cleaning and disinfecting.

Mrs. Patrick Reid has subscribed £500 to the Adelaide Hospital, Dublin, in memory of her late husband.

It is to be hoped that next year the Nonconformists of Savernake will hold collections for the Cottage Hospital.

The Duke of Westminster has sent a cheque for £100 to the National Hospital for the Paralysed, Queen's-square.

Dr. R. Saundby has published his lectures on "Bright's Disease" which he delivered last year at Birmingham.

Medical students at Dundee have the advantage of clinical instruction at the asylum as well as at the infirmary.

Mrs. Angiola Ortelli is superintending the domestic arrangements of the Italian Hospital founded by her husband.

At the annual meeting of University College Hospital authorities it was decided not to rebuild till £30,000 was promised.

The Archbishop of Canterbury will preside at the festival dinner in aid of the National Hospital, Queen's-square, on May 23.

The London street ambulance system arranged by the Hospitals Association will be in working order within two months.

St. Raphael's, Worthing, is the latest addition to the hospitals and convalescent homes which provide for cases of consumption.

An examination for twelve appointments as surgeon in Her Majesty's Indian Medical Service will be held in London in August, 1889.

Mr. W. T. Wing, lately deceased, has left £1,000 to the City of London Hospital for Diseases of the Chest, and to the Ipswich Hospital.

The anniversary festival of the British Orphan Asylum, Slough, was held at the Hotel Metropole last week. Several ladies were present.

A man named Patrick Hanley died lately at the Clonmel Union Hospital. The nuns afterwards found 1,498 dollars concealed in his clothes.

Dr. Charles Booth considers that now the Chesterfield Hospital for accidents has such a large endowment fund, it might make room for medical cases.

The sum of £8 has been received for Fairford Cottage Hospital from Mr. Harry Wakefield by the sale of photographs. Amateur photographers please take the hint.

Mr. Thomas Hollingworth, late of Turkey Court, Maidstone, has left by his will £1,000 each to the West Kent General Hospital and the County Ophthalmic Hospital.

Sir Lyon Playfair presided at the annual meeting of the Royal Normal College for the Blind. The receipts last year amounted to £14,138; the expenditure to £13,288.

According to an official return made for the Austrian Government, the number of practitioners who practise homœopathy is about 1·6 per cent. of the total number of recognised practitioners.

A series of letters appearing in the *Isle of Wight Advertiser* try to prove that the Royal National Hospital is a standing injury to the town of Ventnor. We wonder the editor prints such rubbish.

Louisville is celebrated for its institutions and charities; in medicine it is especially famous. It has four medical colleges, one college of dentistry, one of pharmacy, and one of the latter for women.

The new Victoria Hospital, Hereford, of which the Countess of Chesterfield laid the foundation-stone last December, is rapidly approaching completion, and will be ready to receive patients this summer.

The Lord Mayor has consented to preside at public meetings in aid of the penny-a-week plan on April 13 and 27. Lord Randolph Churchill, Sir Charles Russell, and others have been interested in this fund.

The Bishop of Ripon delivered a lecture at Grosvenor House on April 6 in aid of the Paddington Green Children's Hospital. The lecture was on "Dante," and there was a large and distinguished audience.

Dr. E. J. Brady has received a promise from two gentlemen of £5 each to the funds of the Coombe Lying-in Hospital and Guinness Dispensary, provided eighteen similar sums be contributed within two months.

Dr. Hack Tuke has an interesting article in the current number of *Brain* on "Hallucination and the Subjective Sensations of the Sane." If it were not a paradox, we should say Dr. Tuke proves even the sane to be insane.

The newly-elected and re-nominated members of the Metropolitan Asylums Board have held a private meeting. Sir Edwin Galsworthy and Mr. J. G. Talbot, M.P., were re-elected chairman and vice-chairman for the three years ensuing.

### Amusements and Relaxation.

### ACROSTIC COMPETITION.

As so few competitors have entered for the above competition, we have decided to discontinue it until the autumn, and to run the Word Competition only.

### Word Competition.

At the request of many of our readers we have decided to start another weekly Word Dissection, commencing April 6th, and ending June 29th.

Three Prizes of 15s., 10s., 5s., will be given for the largest number of words derived from the word set for dissection.

Proper names, abbreviations, foreign words, words of less than four letters, and repetitions are barred; plurals, and past and present participles of verbs, are allowed. Nuttall's *Standard* dictionary only to be used.

N.B.—All words sent in must be arranged *alphabetically*, with correct total affixed.

The word for dissection for this, the second week of the quarter, being

"S T A N L E Y."

### Answers.

| No. 26. Double Acrostic. | No. 27. Arithmorem. |
|---|---|
| J eptha H | I liad |
| E li A | R im |
| N eve R | E xodus |
| N ikolae V | L and |
| E ssenc E | A lms |
| R ob Ro Y | N ilotic |
| | D ingle |

### Marks for Solution of Puzzles.

Total Marks.—Flora (2), 15; Mater (1), 11; Scrooge (2), 15; Patience 4; Lady Clara (2), 16; Ruffles, 9; Lauriston, 4; Reldas, (2), 11; Jenny Wren, 5; Condorcet, 2; Tinie, 2; Daffodil, 1.

[*The Figures in parentheses are Marks for the week ending April 4.*]

RESULTS OF SECOND QUARTERLY PRIZE COMPETITION FOR ORIGINAL PUZZLES.

*First Prize* (value £1 1s.), Patience (Miss Calise, Newbury, Berks).

*Second Prize* (15s.), Tinie (Miss Todd, Newbury, Berks).

Third Prize (10s. 6d.), Cotonopolis (Miss M. L. Grundy, Pendleton).

Fourth Prize (7s. 6d.), Lightowlers (Miss C. Chadwick, 98, Huskisson-street, Liverpool.

Fifth Prize (5s.), Prior (Mr. P. Prior, 1, Jesus-terrace, Cambridge).

RESULTS OF SECOND QUARTERLY COMPETITION FOR SOLUTION OF PUZZLES.

*First Prize* (value 15s.), Lady Clara (Mrs. F. W. Hume, Oaklands, Addiscombe).

*Second Prize* (10s. 6d.); a tie between Flora (Miss F. Grundy, 23, Leaf-square, Pendleton), and Scrooge (Miss C. S. Mills, 7, Charles-street, Pendleton).

*Third Prize* (5s.); another tie between Mater (Mrs. Reeves, Barton-under-Needwood), and Reldas (Miss Sadler, Norland-square).

The *Prize Editor* will be glad to hear from the winners of the second and third prizes at once if they would prefer to divide the prizes, or have a test Acrostic set to divide the ties.

N.B. Prize-winners are requested to send in the names of the books they wish for to the "Prize Editor."

### Conditions.

I. Every paper sent in must be clearly written, and one side only must be used. All competitors must mark at the head of their papers the number of their competition.

II. Each paper must be signed by the author with his or her real name and address. A *nom de plume* may be added if the writer does not desire to be referred to by us by his real name. In the case of all prize-winners, however, the real name and address will be published.

III. All communications relating to prize competitions should be addressed to "The Prize Editor, The Hospital, 139 and 140, Salisbury-court, London, E.C." Competitors are requested not to include in letters, etc., to the Prize Editor communications on matters of other business. All letters must be fully prepaid.

IV. The Prize Editor of The Hospital is the sole judge of the competitions, and his decision is in all cases final and without appeal.

V. The Prize Editor reserves the right to publish any paper sent in, whether it receives a prize or not. He does not hold himself responsible for any MSS. sent, nor can he undertake to return rejected competitions.

